# Buffalo Medical and Surgical Association

**Published:** 1883-03

**Authors:** Clayton M. Daniels

**Affiliations:** Secretary


### Society Reports.

### BUFFALO MEDICAL AND SURGICAL ASSOCIATION.

*Regular Meeting, February 6th, 1883.*

President, Dr. A. R. Davidson, in the Chair. Dr. C. M. Daniels, Secretary.

Present—Drs. Barrett, Coakley, Fowler, Heath, Warren, Brecht, Runner, Moody, Cronyn, Granger, Hubbell, Kamerling, Bartlett, Burghardt, Keene, and by invitation, Dr. Hayd and Mr. F. R. Campbell.

Applications for membership were received from Drs. G. W. York and Jacob Frank, who were duly elected.

Dr. Granger read a paper entitled "Paresis."

*Discussion*—Dr. Cronyn said Dr. Granger had most elaborately dealt with his subject, the disease itself being one in which the individual always imagines himself pleasantly situated, having great wealth and offering to divide with those encouraging his numerous fancies; related a case which appeared very suddenly, following an epileptiform convulsion, hemiplegia being present which was partially recovered from; thought that alcoholism sometimes simulated it very closely.

Dr. Bartlett had seen but few cases, but believed that the longer insanity was studied the more structural changes would be found; related a case where a prominent man, afflicted with "paresis" in early stages, came to him and said people accused him of being drunk. Speaking of the exalted idea so generally noticed, he had thought that perhaps the undue mental effort in the development of grand schemes, &c., might tend to produce "paresis" itself.

Dr. Hubbell wished to acknowledge the interest he had taken in the paper, especially as he had a typical case now under his care, which was passing through the various stages as cited by the essayist; thought treatment was only palliative; had used strychnia, faradization, &c.

Dr. Heath thought the paper the most interesting he had heard on the subject. Referring to the possible syphilitic origin of "paresis," he thought that much was attributed to syphilis unjustly; thought in "paresis" it was hard to determine which comes first, the mental or physical troubles, and that it compared intimately with sclerosis of the cord.

Dr. Granger in closing said that, regarding sclerosis, he thought that it was frequently associated also that the condition was not true paralysis, but a want of co-ordination as the disease progresses. True paralysis may follow; post-mortem clots have been found upon the brain.

Dr. Barrett asked if Dr. Granger had discovered any pathological changes.

Dr. Granger replied that the differences were wide in various conditions.

Dr. Heath asked if it was, strictly speaking, a disease by itself.

Dr. Granger replied that it was now so considered.

Dr. Hayd asked if the skin or tendon reflex showed any increase of irritability.

Dr. Granger thought that tendon reflex was of no diagnostic importance.

Dr. Cronyn said that it was only of value when the cord had become engaged.

Dr. Granger remarked that the cord was involved more frequently than was formerly supposed.

Dr. Hubbel asked that the essayist would recommend as to treatment.

Dr. Granger said the cases were incurable; that treatment was of little value and only to prolong life, which certainly seemed undesirable, as in the last stages the patients are reduced to the most pitiable conditions.

*Voluntary Communications*—Dr. Bartlett made a few remarks and presented a specimen of a placenta, where patient had passed through nine confinements normally; then the tenth, eleventh and twelfth children were all born dead; hemorrhage severe ; comatose condition followed. The placenta shown was from the twelfth confinement and presented a peculiarly condensed, pale condition. It was carefully examined by members of the association.

Dr. Cronyn thought the abnormalities were not so great as it was at first supposed, and that the death of the children might have been from other causes.

Dr. Bartlett replied that to him it seemed a matter of a good deal of importance, and that he knew of a case where twelve children were all born dead, and in his own experience where four were dead and another lived but a short time.

Dr. Cronyn, in replying to Dr. Bartlett, related a case where he was called to see a lady having acute bronchitis, was seven months pregnant, in four or five days, gave premature birth to a boy ; had previously given birth to six girls. Within the last ten days he had been called to see her, and found she had pneumonia and was again pregnant and premature birth of a boy again followed. He asked Dr. Bartlett why should this occur to a patient having an acute affection of the lungs and both children be boys.

Dr. Bartlett said the comparison was hardly fair, as the full quota of boys was not yet reported.

*Prevailing Diseases*—Dr. Cronyn had noted a great deal of follicular tonsillitis and diphtheria. Dr. Bartlett had a similar report and added " some scarlatina."

Adjourned. CLAYTON M. DANIELS, *Secretary.*

